# Electroconvulsive seizures regulate various stages of hippocampal cell genesis and mBDNF at different times after treatment in adolescent and adult rats of both sexes

**DOI:** 10.3389/fnmol.2023.1275783

**Published:** 2023-10-30

**Authors:** Sandra Ledesma-Corvi, M. Julia García-Fuster

**Affiliations:** ^1^IUNICS, University of the Balearic Islands, Palma, Spain; ^2^Health Research Institute of Balearic Islands (IdISBa), Palma, Spain; ^3^Department of Medicine, University of the Balearic Islands, Palma, Spain

**Keywords:** neurogenesis, BrdU, Ki-67, NeuroD, BDNF, mTOR, ERK1/2

## Abstract

Electroconvulsive therapy, a fast-acting option for treatment-resistant depression, is modeled at the preclinical level through the induction of electroconvulsive seizures (ECS) in rodents. Recent studies from our group proved sex- and age-differences in the antidepressant-like response elicited by ECS in rats; while an antidepressant-like response was observed in male adolescent and adult rats (although with greater efficacy in adulthood), the same parameters rendered inefficacious in females of any age. To better understand the potential sex differences taking place at the molecular level that might be mediating these behavioral disparities, we evaluated the impact of a repeated treatment with ECS (95 mA for 0.6 s, 100 Hz, 0.6 ms) in adolescent and adult rats of both sexes. Several hippocampal markers of neuroplasticity, commonly regulated by most antidepressants, such as those of neurogenesis (cell proliferation, neurogenic differentiation, long-term cell survival) or mBDNF and associated signaling (e.g., mTOR and ERK1/2) were evaluated at different time-points after treatment (1-, 8-, 15- and up to 30-days post-treatment). The main results demonstrated that ECS improved the survival rate of new cells born in the dentate gryus before treatment. Moreover, ECS increased cell proliferation and neurogenic differentiation at different times post-treatment, paired with persistent increases in mBDNF, observed long after treatment. In general, effects were different for each sex and varied with the age of the animal (adolescent vs. adulthood). The present study is the first-one to demonstrate that such persistent molecular changes induced by ECS in hippocampus, some of them observed up to 30-days post-treatment, also occurred in female rats and adolescence. Although these molecular changes could not justify the lack of ECS efficacy described by these same parameters of ECS in female rats (vs. male rats), they proposed certain beneficial effects common to both sexes, and age periods studied, opening the avenue for further studies. Based on these neurochemical effects, ECS should have displayed similar efficacies for both biological sexes. Therefore, the reason behind these disparities should be further explored to better translate efficacious treatments specific and/or personalized for each sex to the clinic.

## 1. Introduction

Electroconvulsive therapy (ECT) is a non-pharmacological treatment based on altering neural circuitries through an electrical stimulation that produces a generalized seizure, which is known to induce safe, fast-acting and long-lasting antidepressant-like responses in adult patients with treatment-resistant depression (e.g., Trifu et al., 2021; Subramanian et al., 2022). However, its use for adolescent depression, whose rates are rising over these past years (reviewed by Keyes and Platt, 2023), is almost inexistent, even though prior reports suggested that it is safe and efficacious for this age group (e.g., Castaneda-Ramirez et al., 2023). Consequently, the generation of new high-quality data, improved experience and additional knowledge for child and adolescent psychiatrists would be key to increase the clinical use of ECT for adolescents (Licht et al., 2023). Moreover, besides age, biological sex should be considered when defining the specific parameters that will be needed to induce efficacy (Sackeim et al., 1987; Rasimas et al., 2007; Peterchev et al., 2010; Lemasson et al., 2018; Güney et al., 2020; Blanken et al., 2023), since prior reports described that women required a lower charge to induce an optimal seizure than men at the same age, and for both sexes the charge needed increased with age (Salvador Sánchez et al., 2017).

In this context, a recent study from our group demonstrated age- and sex-specific differences in the antidepressant-like potential of repeated electroconvulsive seizures (ECS), a preclinical model of ECT in naïve and maternally deprived rats (García-Cabrerizo et al., 2020). While the treatment exerted antidepressant-like effects when administered during adolescence or adulthood in male rats (although with a shorter period of effectiveness for adolescence), in female rats it rendered deleterious in adolescence and/or ineffective in adulthood (ECS parameters: 95 mA for 0.6 s at a frequency of 100 Hz square wave pulses, pulse width 0.6 ms, 5 days, 1 shock/day; see García-Cabrerizo et al., 2020 for more details). The lack of efficacy observed in females for the 95-mA intensity dose was replicated in a separate study (Ledesma-Corvi and García-Fuster, 2023), in which we also demonstrated that decreasing the intensity used for ECS treatment to 75 or 55 mA, was indeed capable of inducing an antidepressant-like response in adult female rats, in line with the observations from the clinical data (Salvador Sánchez et al., 2017), while still no effects were observed for female adolescent rats (Ledesma-Corvi and García-Fuster, 2023), reinforcing clear age-related differences in treatment response.

Against this background, the goal of the present study was to ascertain potential molecular correlates that could help us better understand the behavioral sex- and age-disparities elicited by ECS, since the exact mechanism of action by which it induces an antidepressant-like response has not been completely elucidated (see Commentary by Krishnan, 2016). Interestingly, the induction of hippocampal neuroplasticity, with processes such as neurogenesis (e.g., Madsen et al., 2000; Malberg et al., 2000; Scott et al., 2000; reviewed by Segi-Nishida, 2011; Jonckheere et al., 2018; Ueno et al., 2019) and/or the activation of neurotrophic factors (i.e., BDNF and subsequent signaling via TrkB receptors, e.g., Björkholm and Monteggia, 2016; Casarotto et al., 2021; Madjid et al., 2023) have been studied for many years now in the context of being key transducers of antidepressant effects, and with various results pointing at their clear contribution to the beneficial response of ECS (recently reviewed by An and Wang, 2022). From the well documented and extensive summary recently presented in An and Wang (2022) on how ECS modulates neurogenesis and neurotrophy in hippocampus, the take home messages concluded that repeated ECS increased cell proliferation and BDNF in a dose- and time-dependent manner (bigger effects with a repeated vs. acute paradigm); the increase in cell proliferation correlated with the amount of ECS administered; and the newly born cells survived for a long time. However, most, if not all, of these previous studies, did not considered sex as a biological variable, causing a gap in the literature regarding the potential effects of ECS at the neurochemical level in female rodents. Also, all prior studies were almost exclusively done in adult male rodents, with little to inexistent data for adolescence (e.g., Jonckheere et al., 2018; An and Wang, 2022). In this regard, we have performed two recent studies proving that repeated ECS increased cell proliferation and/or vastly boosted young neuronal survival in a course manner and in a similar way for both sexes and independently of age (García-Cabrerizo et al., 2020; Ledesma-Corvi and García-Fuster, 2023).

**Figure 1:**
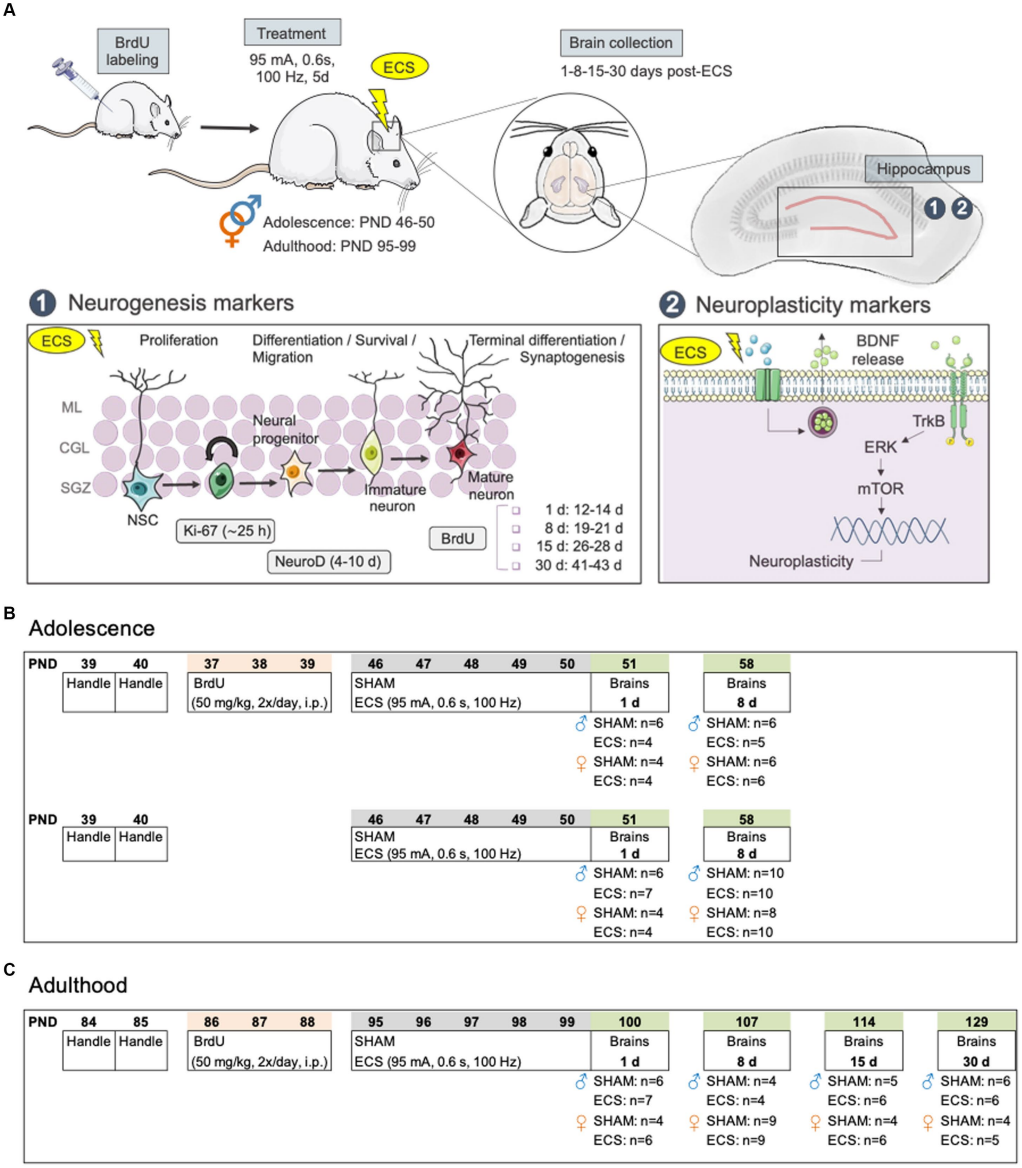
**(A)** Diagram of hippocampal markers selected for evaluation: 1. Neurogenesis markers (i.e., Ki-67, NeuroD, BrdU); 2. Other neuroplasticity makers (i.e., BDNF, p-mTOR/mTOR, p-ERK1/2/ERK1/2). **(B)** Experimental timeline in adolescent rats. **(C)** Experimental timeline in adult rats. BDNF, brain-derived neurotrophic factor; BrdU, 5-bromo-2′-deoxyuridine; ECS, electroconvulsive seizures; ERK, extracellular signal-regulated kinase; d, day; mTOR, mammalian target of rapamycin; NSC, neural stem cells; PND, post-natal day; TrkB, tropomyosin receptor kinase B.

Against this background, the present study aimed at further characterizing the effects induced by ECS in hippocampus by sex and age, by complementing our prior data (García-Cabrerizo et al., 2020; Ledesma-Corvi and García-Fuster, 2023) and studying the impact of repeated ECS on the different stages of the neurogenic process: cell proliferation, neurogenic differentiation, as well as long-term survival of cells born before treatment, labeled with BrdU (see Figure 1A). Moreover, since following ECS treatment BDNF levels paralleled the regulation of adult neurogenesis (reviewed by An and Wang, 2022), we also aimed at ascertaining the regulation of the mature form of BDNF (mBDNF; linked with antidepressant-like effects), together with certain of its downstream signaling partners whose regulation is pretty much unexplored in this context (i.e., p-ERK1/2 or p-mTOR; e.g., Gangarossa et al., 2015), but appeared as common molecular mechanisms of several rapid-acting antidepressants (reviewed by Chen et al., 2023). We performed an extensive characterization of the regulation of these molecular events following repeated ECS treatment in male and female rats at two key age periods (adolescence vs. adulthood) while incorporating a long-term evaluation at different time-points after treatment: 1- and 8-days post-treatment for adolescent rats, and 1-, 8-, 15- and up to 30-days post-treatment for adult rats.

## 2. Methods

### 2.1. Animals

For the present study, we used a total of 100 adolescent (54 males and 46 females; Figure 1C) and 91 adult (44 males and 47 females; Figure 1C) Sprague–Dawley rats that were bred in the animal facility at the University of the Balearic Islands. During all procedures, rats were housed in groups of 2 to 4 rats per cage under controlled environment settings (T = 22°C; humidity = 70%; light/dark cycle: 12:12 h; light on at 8:00 h) and unrestricted access to a standard diet and water. All procedures were approved by the Local Bioethical Committee (University of the Balearic Islands) and the regional Government (Conselleria Medi Ambient, Agricultura i Pesca, Direcció General Agricultura i Ramaderia, Govern de les Illes Balears) following ARRIVE guidelines (Percie du Sert et al., 2020), the EU Directive 2010/63/EU for animal experiments, and the Spanish Royal Decree 53/2013. Efforts were made to minimize the number of rats used and their suffering. In this context, animals were handled by the experimenter prior to any procedures to reduce future stress and/or suffering. Moreover, in terms of reducing and reusing animals, some of these rats were previously used to behaviorally characterize the differences in the antidepressant-like responses induced by ECS in male and female rats, both during adolescence and adulthood (see García-Cabrerizo et al., 2020; Ledesma-Corvi and García-Fuster, 2023).

### 2.2. BrdU pretreatment for cell survival analysis

As specified in Figures 1B,C, a group of adolescent rats and all adults received 5-bromo-2′-deoxyuridine (BrdU, 2 × 50 mg/kg, one pulse every 12 h, i.p.; Calbiochem, United States) for 3 days (PND 37-39, Figure 1B or PND 86-88, Figure 1C) following a standard procedure (e.g., see García-Fuster et al., 2010; García-Cabrerizo et al., 2018, 2021). As previously detailed in García-Fuster et al. (2010), this protocol prevents marker dilution due to cell division (Dayer et al., 2003) and labels newly generated cells that will differentiate and incorporate within hippocampus (Kempermann, 2002; Kempermann et al., 2004) following 28 days of survival (Brown et al., 2003). Moreover, this paradigm of BrdU administration was given 4 days before treatment started (Thomas et al., 2007), so we could later study the impact of repeated ECS on the survival of these cells as measured 1-, 8-, and up to 15- or 30-days post-treatment (Figure 1). At these time-points of study BrdU +cells were between 12–14, 19–21, 26–28 or 41–43 days old, respectively (Figure 1A). Note that for adolescent rats, these evaluations were only performed 1- and 8-days post-treatment, so rats were in the adolescent period at the time-point of analysis (<PND 60; see Figure 1B).

### 2.3. Electroconvulsive seizures (ECS) treatment and tissue collection

In a series of independent experiments, randomly allocated rats from each age and sex groups (see Figures 1B,C) were exposed to daily ECS sessions (95 mA for 0.6 s at a frequency of 100 Hz square wave pulses, pulse width 0.6 ms) using a pulse generator (ECT Unit 7,801; Ugo Basile, Italy) via earclip electrodes or were connected to the electrodes with no electrical current (SHAM) over a 5-day period (PND 46-50 or PND 95-99 for the adolescent or adult window of treatment exposure). The intensity of ECS was chosen based on prior studies from our group (García-Fuster and García-Sevilla, 2016; García-Cabrerizo et al., 2020), capable of inducing the expected daily seizures (i.e., tonic and clonic phases). Brains were collected following rapid decapitation at different time-points after treatment: 1- and 8-days post-treatment for adolescent rats (Figure 1B), and also up to 15- and 30-days post-treatment for adults (Figure 1C). From each collected brain, the left half-hemisphere was quickly frozen in isopentane at-30°C, while the right hippocampus was freshly dissected and fast-frozen in liquid nitrogen. All samples were then stored at-80°C until further use.

### 2.4. Hippocampal neurogenesis markers by immunohistochemistry

Tissue was prepared for immunohistochemistry analysis, by cryostat-cutting the whole extent of the left hippocampus (from −1.72 to −6.80 mm from Bregma) in 30 μm sections that were slide-mounted and frozen at −80°C (García-Fuster et al., 2010; García-Cabrerizo et al., 2018, 2021). In terms of the markers used for neurogenesis analysis, BrdU labeling was utilized to evaluate how ECS affected the survival of cells that were born prior to treatment (Figure 1A). This analysis was done for male and female adolescent and adult rats. Moreover, Ki-67 (an intrinsic marker of cell proliferation) and NeuroD (which labels neurogenic differentiation; Figure 1A) were assessed exclusively in adult female rats, since their regulation in adolescent rats of both sexes as well as in adult males was previously performed (see García-Cabrerizo et al., 2020; Ledesma-Corvi and García-Fuster, 2023). These markers have been previously reliably used to label how a particular treatment might affect the different stages of hippocampal neurogenesis by immunohistochemistry (for further details on marker selection see García-Fuster et al., 2010).

For each particular procedure and molecular marker, we performed a separate immunohistochemistry experiment with 3 slides per animal. Each slide contained every 8th tissue section from the anterior-middle-posterior parts of hippocampus respectively, providing 8 tissue-sections/slide for a total of 24 sections/rat for each marker analyzed (García-Fuster et al., 2010, 2011; García-Cabrerizo et al., 2018, 2020, 2021). The day of the immunohistochemistry procedure, tissue was post-fixed in 4% paraformaldehyde and exposed to several steps such as antigen retrieval, blocking in peroxidase solution and BSA, including the incubation with one of the following primary polyclonal antibodies: rabbit anti-BrdU (1:40000) or anti-Ki-67 (1:20000) (both generously provided by Drs. Huda Akil and Stanley J. Watson, University of Michigan, MI, United States), and goat anti-NeuroD (1:10000; R&D Systems Bio-Techne, MN, USA). The next steps included a series of sequential incubations, including that with a biotinylated anti-rabbit or anti-goat secondary antibody (1:1000, Vector Laboratories, CA, United States), an Avidin/Biotin complex (Vectastain Elite ABC kit; Vector Laboratories), the chromogen 3,3′-diaminobenzidine (DAB) (with nickel chloride for NeuroD) for signal detection, and tissue counterstaining with cresyl violet, but only for BrdU and Ki-67 labeling. Finally, all tissue was dehydrated in graded alcohols, immersed in xylene and cover-slipped using Permount® mounting medium.

Prior to counting, all slides were coded to ensure that all immunostained +cells were blindly quantified in regards to the pertaining experimental group. Labeled cells were counted in the dentate gyrus while focusing through the thickness of the tissue section with a Leica DMR light microscope (63x objective lens and 10x ocular lens; total magnification of 630x). The total number of +cells in each slice was multiplied by the sampling factor 8 (since every 8th section through the entire hippocampus was counted; e.g., Malberg et al., 2000; Malberg and Duman, 2003) to provide an estimate of the total number of +cells per labeled marker and rat hippocampi at each condition and/or time-point of analysis (see our prior publications following the same procedure for over 10 years; e.g., García-Fuster et al., 2010, 2011; García-Cabrerizo et al., 2020, 2021). Note that for BrdU immunohistochemistry, some adult rats were missing from the final analysis (1 for male-SHAM-1 d; 2 for male-ECS-1 d; 1 from female-ECS-1 d; and 1 from female-SHAM-8 d) due to incorrect tissue labeling.

### 2.5. Neuroplasticity markers by western blot

The right hippocampus was prepared for western blot analysis, by homogenizing tissue as previously described (e.g., García-Cabrerizo et al., 2015). Immunodensities of the selected neuroplasticity markers (i.e., mBDNF, p-ERK1/2 and p-mTOR) were assessed by separating 40 μg of total homogenates by electrophoresis on 10–15% SDS-PAGE mini-gels (Bio-Rad Laboratories, CA, United States). Proteins were then transferred to nitrocellulose membranes and incubated overnight (at 4°C) with a blocking solution containing the appropriate primary antibody: anti-BDNF (1:2500; Abcam, Cambridge, United Kingdom); anti-p-ERK1/2 (p44/ p42) (1:1000; Cell Signaling, MA, USA); anti-p-mTOR (Ser2448) (1:1000; Cell Signaling). The next day, membranes were incubated with the appropriate secondary antibody (anti-rabbit or anti-mouse IgG linked to horseradish peroxidase; 1:5000 dilution; Cell Signaling). Immunoreactivity was detected by incubating membranes with ECL reagents (Amersham, Buckinghamshire, United Kingdom) and placing them in contact with an autoradiographic film (Amersham ECL Hyperfilm) for 1–60 min. Films were then analyzed by densitometric scanning (GS-800 Imaging Calibrated Densitometer, Bio-Rad) and percent changes in immunoreactivity were calculated for each sample in relation to SHAM controls for that particular membrane (i.e., average of at least 3-4 controls per membrane). Each sample was loaded at least in 3-5 different gels (replicates), and the mean value was used as a final estimate of the specific protein content. Membranes from the blots for p-ERK1/2 and p-mTOR were stripped and reprobed with antibodies labeling the total protein forms: anti-ERK1/2 (1:1000; Cell Signaling) and anti-mTOR (1:1000; Cell Signaling), so results were expressed as the ratio of phosphorylated vs. total forms (p-EKR1/2/ERK1/2 and p-mTOR/mTOR). Also, ß-actin (clone AC-15) (1:10000; Sigma–Aldrich, MO, United States) served as a loading control since it was not altered by any treatment condition.

### 2.6. Statistical analysis

Data analysis and graph plotting was done with GraphPad Prism 10 for Mac OS (GraphPad Software, CA, USA), in accordance with the guidelines for displaying data and statistical methods in experimental pharmacology (e.g., Curtis et al., 2018; Michel et al., 2020). In all graphs, bars represent the mean values of the particular marker under evaluation ± the standard error of the mean (SEM), while symbols represent individual values for each rat. The regulation of various stages of hippocampal neurogenesis (BrdU, Ki-67 or NeuroD +cells) and other neuroplasticity markers (mBDNF, pERK1/2/ERK1/2, p-mTOR/mTOR) by ECS for each sex, age and time point of analysis (1-, 8-, 15- or 30-days post-treatment) was evaluated through Student’s *t* test comparisons (SHAM vs. ECS). These variables were not included in the statistical analysis, since brains were collected at different time points throughout the year from independent experiments that were separately designed for each sex and age. Therefore, the particular conditions at each time point of sample collection and/or sample processing for the molecular analyzes might induce basal differences in the regulation of these molecular markers in the control groups (see for example our prior study, García-Fuster and García-Sevilla, 2016, and the references within). The level of significance was set at *p* ≤ 0.05. The data that supports the findings of this study will be available upon reasonable request to the corresponding author.

## 3. Results

### 3.1. ECS improves the survival of hippocampal newly-generated cells, born before treatment, in adolescent and adult rats

**Figure 2:**
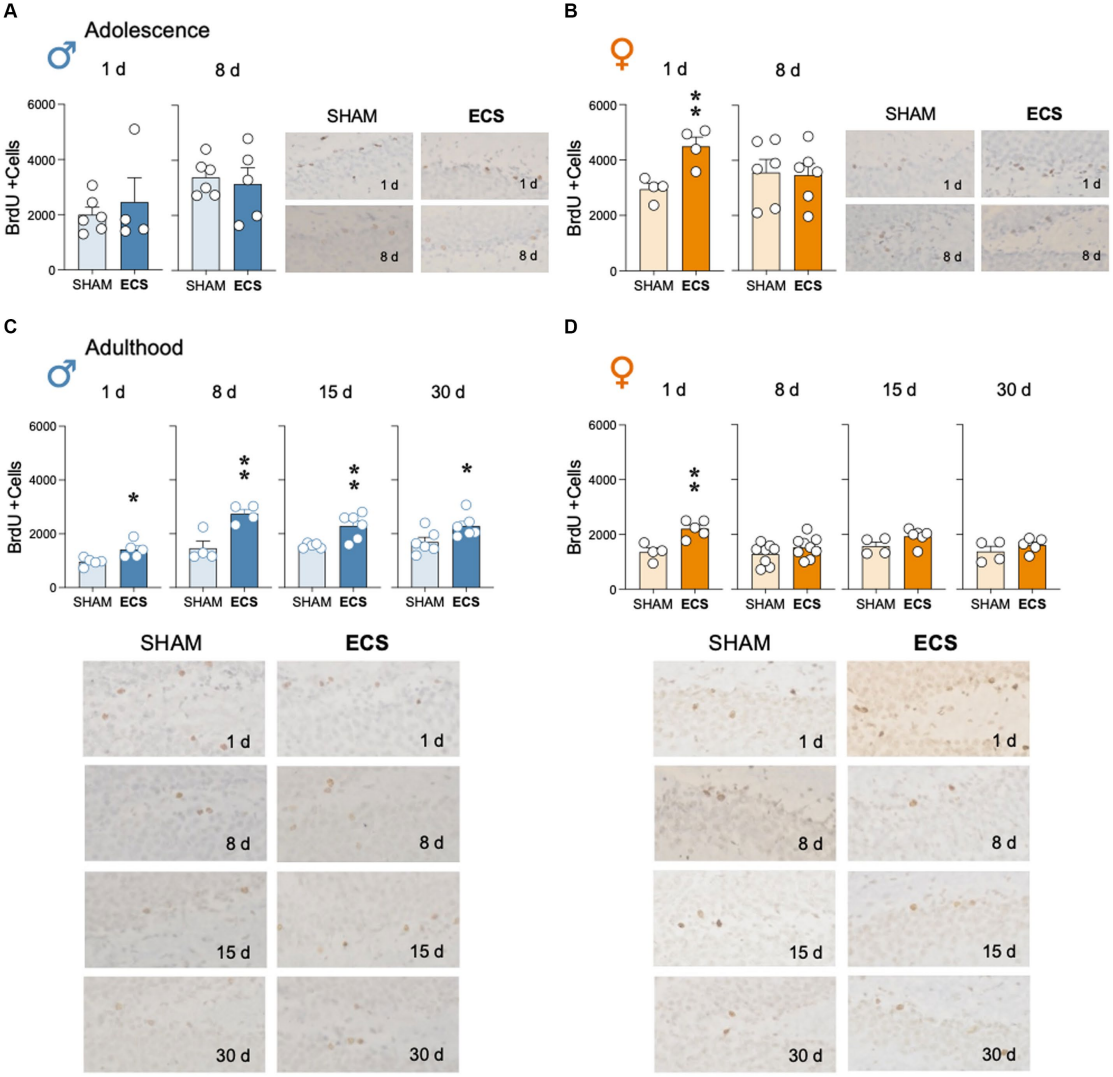
Modulation of hippocampal cell survival (BrdU +cells) by repeated ECS treatment in male **(A)** and female **(B)** adolescent rats as measured 1-day and 8-days after treatment, or in male **(C)** and female **(D)** adult rats as measured 1-, 8-, 15- and up to 30-days post-treatment. Data represent mean ± SEM of BrdU total estimated +cells in the dentate gyrus as measured by immunohistochemistry analysis. Individual values are shown for each rat (symbols). Pair comparisons for each sex and age of study were done by Student’s *t*-tests: *p < 0.05 and **p < 0.01 vs. SHAM rats. Representative images of BrdU + cells (dark brown labeling in a lighter granular layer background), taken with a light microscope and quantified with a 63x objective lens, are shown next to the bar graphs.

Repeated ECS treatment during adolescence did not alter the number of surviving BrdU +cells as measured 1-day (age of cells: 12-14 days; *t* = 0.58, *df* = 8, *p* = 0.578) or 8-days post-treatment (age of cells: 19-21 days; *t* = 0.38, *df* = 9, *p* = 0.710; Figure 2A) in hippocampus of male rats. However, in adolescent female rats, ECS increased the survival of BrdU +cells 1-day post-treatment (+1,542 ± 395; *t* = 3.90, *df* = 6, **p = 0.008 vs. SHAM), but not 8-days post-treatment (*t* = 0.15, *df* = 10, *p* = 0.883; Figure 2B).

In adult male rats, ECS increased BrdU +cells surviving in hippocampus at the time of treatment exposure. In particular, BrdU was increased at all time points analyzed, although the number of +cells seem to decrease with time: 1-day (+459 ± 135; *t* = 3.04, *df* = 8, *p = 0.016); 8-days (+1,278 ± 312; *t* = 4.09, *df* = 6, **p = 0.006 vs. SHAM), 15-days (+731 ± 220; *t* = 3.31, *df* = 9, **p = 0.009 vs. SHAM), and up to 30-days post-treatment (+596 ± 247; *t* = 2.41, *df* = 10, *p = 0.037 vs. SHAM; Figure 2C). However, in adult female rats, ECS increased the number of BrdU +cells, but only 1-day post-treatment (+860 ± 209; *t* = 4.11, *df* = 7, **p = 0.005 vs. SHAM), since similar levels of +cells were observed 8- (*t* = 1.38, *df* = 15, *p* = 0.188), 15- (*t* = 1.89, *df* = 8, *p* = 0.095) and 30-days post-treatment (*t* = 1.10, *df* = 7, *p* = 0.310; Figure 2D).

### 3.2. ECS improves the rate of cell proliferation and neurogenic differentiation in hippocampus of adolescent and adult female rats

**Figure 3:**
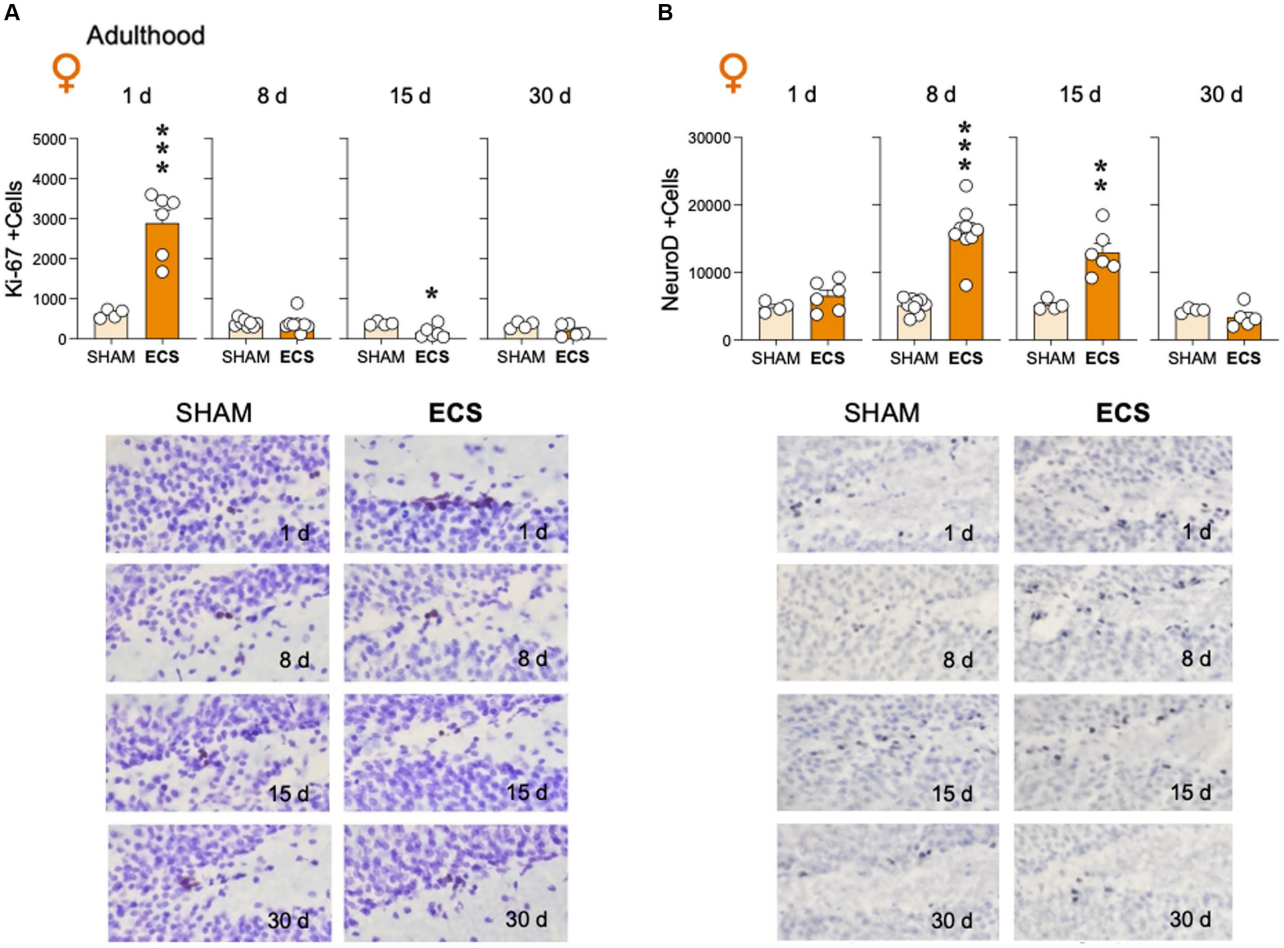
Modulation of hippocampal **(A)** cell proliferation (Ki-67 + cells) and **(B)** neurogenic differentiation (NeuroD +cells) by repeated ECS treatment in female adult rats as measured 1-, 8-, 15- and up to 30-days post-treatment. Data represent mean ± SEM of the number of total estimated +cells in the dentate gyrus as measured by immunohistochemistry analysis. Individual values are shown for each rat (symbols). Pair comparisons for each sex and age of study were done by Student’s *t*-tests: *p < 0.05, **p < 0.01 and ***p < 0.001 vs. SHAM rats. Representative images of **(A)** Ki-67 + cells (dark brown labeling in a blue granular layer background) or **(B)** NeuroD +cells (dark blue labeling in a lighter blue granular layer background), taken with a light microscope and quantified with a 63x objective lens, are shown below the bar graphs.

The present study ascertained how repeated ECS modulated Ki-67 (Figure 3A) and NeuroD (Figure 3B) in adult female rats. The results showed that ECS treatment rapidly increased the number of Ki-67 + cells as measured 1-day post-treatment (+2,264 ± 413; *t* = 5.48, *df* = 8, ***p < 0.001 vs. SHAM), to then normalize 8-days post-treatment (*t* = 0.12, *df* = 14, *p* = 0.907), while followed by a significant reduction 15-days post-treatment (−218 ± 77; *t* = 2.82, *df* = 8, *p = 0.023 vs. SHAM), and another stabilization at 30 days (*t* = 1.57, *df* = 7, *p* = 0.162). On the other hand, ECS modulated NeuroD+ at different time-points after treatment, with no significant increases observed 1-day post-treatment (+1,653 ± 1,159; *t* = 1.43, *df* = 8, *p* = 0.191), a peak expression at 8-days (+11,018 ± 1,340; *t* = 8.22, *df* = 16, ***p < 0.001 vs. SHAM) with maintained high numbers at 15-days (+7,802 ± 1709; *t* = 4.56, *df* = 8, **p = 0.002 vs. SHAM), followed by a normalization 30-days post-treatment (*t* = 1.23, *df* = 7, *p* = 0.259).

### 3.3. ECS increases mBDNF protein content in hippocampus of adolescent and adult rats

**Figure 4:**
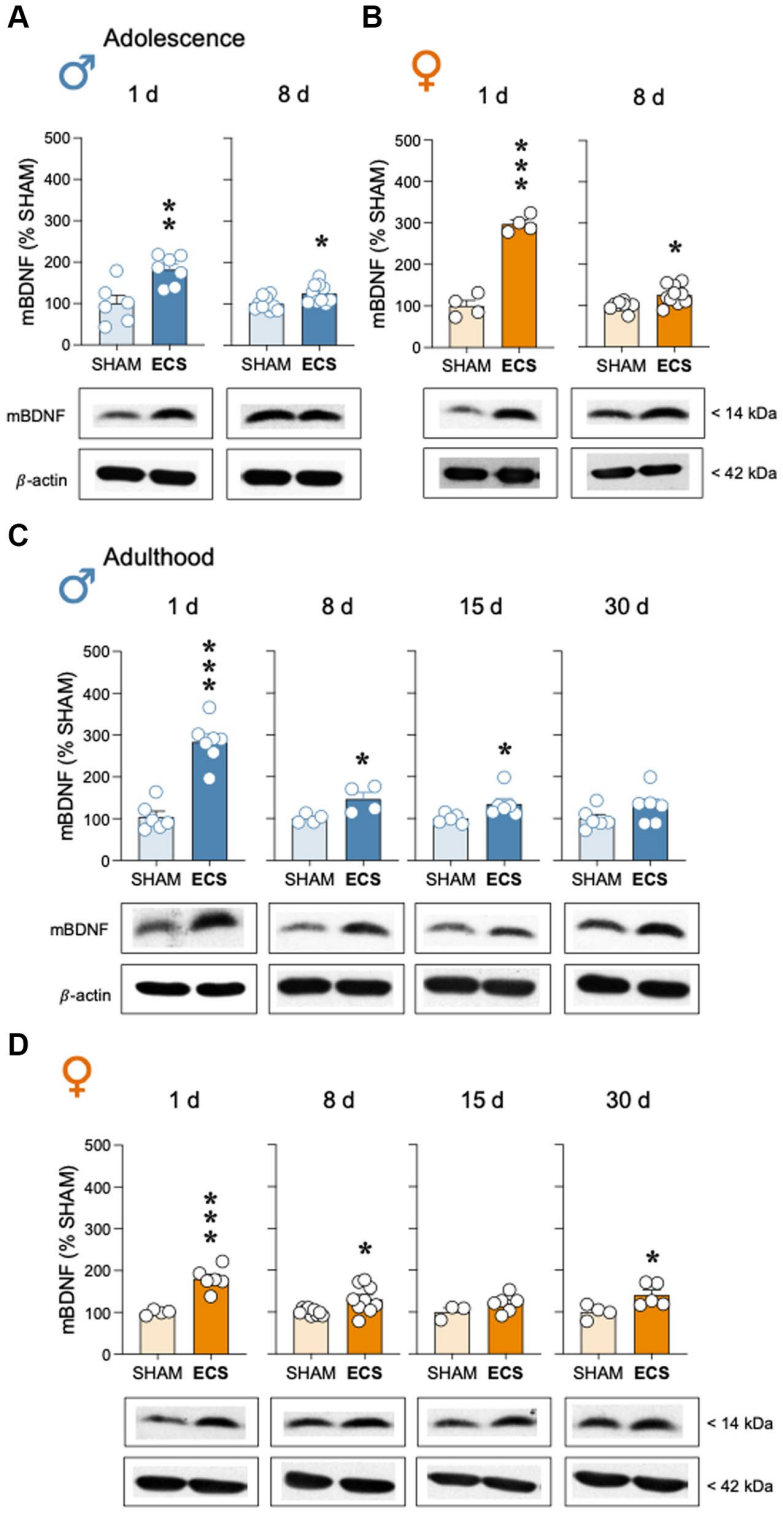
Modulation of hippocampal mBDNF protein content by repeated ECS treatment in male **(A)** and female **(B)** adolescent rats as measured 1-day and 8-days after treatment, or in male **(C)** and female **(D)** adult rats as measured 1-, 8-, 15- and up to 30-days post-treatment. Data represent mean ± SEM of mBDNF protein content expressed as % change vs. SHAM-treated control rats at each particular time and as ascertained by western blot analysis. Individual values are shown for each rat (symbols). Pair comparisons for each sex and age of study were done by Student’s *t*-tests: *p < 0.05, **p < 0.01 and ***p < 0.001 vs. SHAM rats. Representative immunoblots depicting mBDNF and β-actin (as a loading control) are shown below each bar graph.

Adolescent ECS treatment up-regulated mBDNF protein content in hippocampus of male rats, both 1-day (+82 ± 23%; *t* = 3.52, *df* = 11, **p = 0.005 vs. SHAM) and up to 8-days post-treatment (+25 ± 9%; *t* = 2.80, *df* = 18, *p = 0.012 vs. SHAM; Figure 4A); similar results were observed for female adolescent rats (1-day: +197 ± 17%; *t* = 11.88, *df* = 6, ***p < 0.001; 8-days: +26 ± 9%; *t* = 2.89, *df* = 16, **p = 0.011 vs. SHAM; Figure 4B). During adulthood, ECS also increased mBDNF in male (Figure 4C) and female rats (Figure 4D). In particular, in adult male rats, following the increase observed 1-day post-treatment (+179 ± 24%; *t* = 7.33, *df* = 11, ***p < 0.001 vs. SHAM), mBDNF was still increased 8-days (+46 ± 17%; *t* = 2.76, *df* = 6, *p = 0.033 vs. SHAM) and up to 15-days post-treatment (+35 ± 15%; *t* = 2.30, *df* = 9, *p = 0.047 vs. SHAM), with normalized levels at 30 days (+30 ± 19%; *t* = 1.54, *df* = 10, *p* = 0.155; Figure 4C). Similar long-term effects were observed for adult female rats (1-day: +80 ± 14%; *t* = 5.63, *df* = 8, ***p < 0.001; 8-days: +32 ± 11%; *t* = 2.73, *df* = 16, *p = 0.015; 15 days: +21 ± 14%; *t* = 1.41, *df* = 7, *p* = 0.200; 30-days: +41 ± 16%; *t* = 2.61, *df* = 7, *p = 0.035 vs. SHAM; Figure 4D).

**Figure 5:**
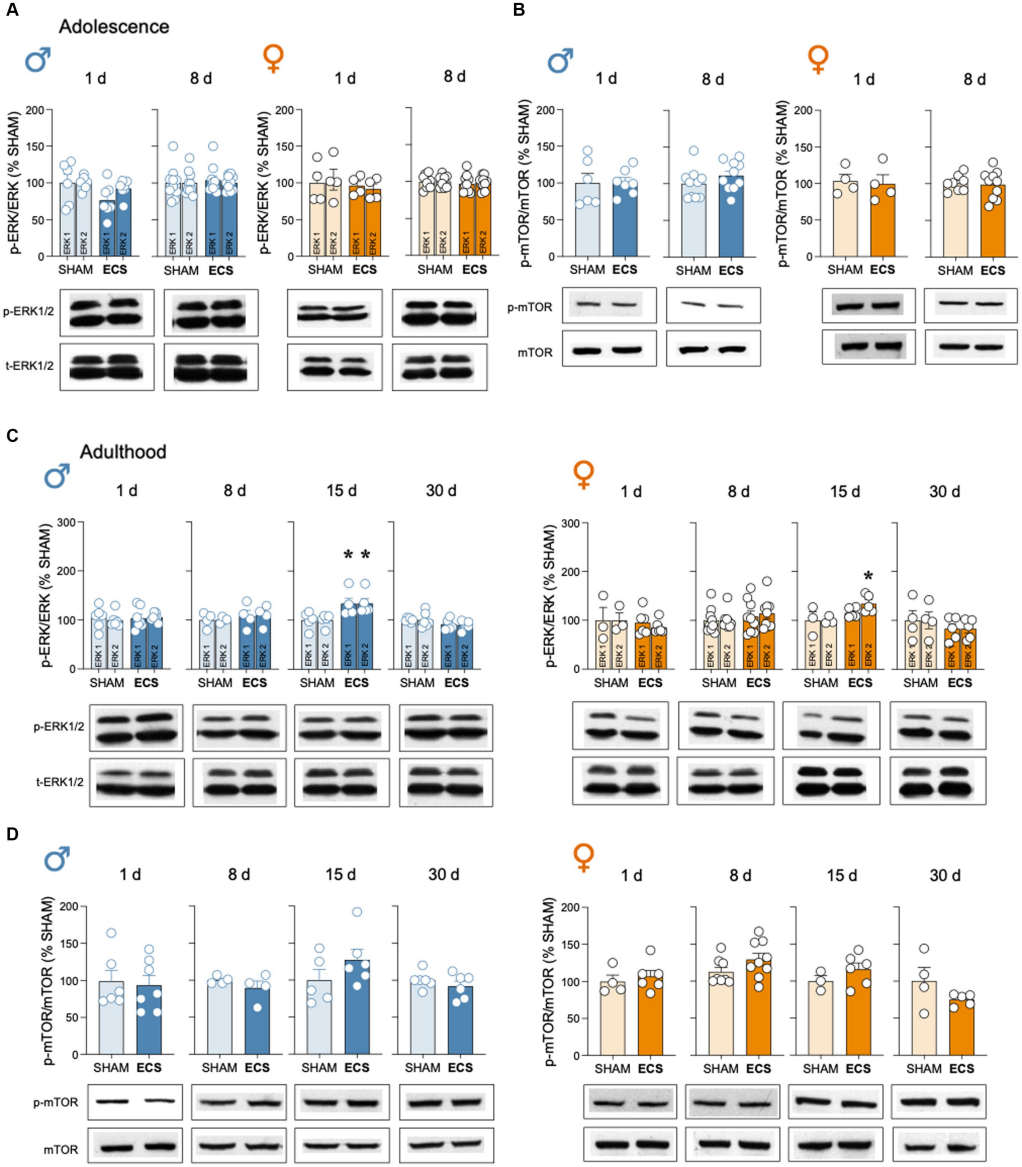
Evaluation of the protein content of certain neuroplasticity markers in hippocampus following repeated ECS treatment by western blot analysis. **(A)** p-ERK1/2/ERK1/2 and **(B)** p-mTOR/mTOR in male and female adolescent rats as measured 1-day and 8-days after treatment. **(C)** p-ERK1/2/ERK1/2 and **(D)** p-mTOR/mTOR in male and female adult rats as measured 1-, 8-, 15- and up to 30-days post-treatment. Data represents mean ± SEM of the ratio between the phosphorylated vs. total form of the protein evaluated and expressed as % change vs. SHAM-treated control rats at each particular time. Individual rates are shown for each rat (symbols). Pair comparisons for each sex and age of study were done by Student’s *t*-tests: **p < 0.01 vs. SHAM rats. Representative immunoblots depicting p-ERK1/2, ERK1/2, p-mTOR and mTOR are shown below each bar graph.

Finally, ECS treatment did not modulate p-ERK1/2/ERK1/2 or p-mTOR/mTOR in hippocampus of male and female adolescent rats at any time points of analysis (Figures 5A,B). However, a moderate increase was observed in p-ERK1/2/ERK1/2 15-days post-ECS treatment in adult rats (Figure 5C), while no changes were observed or p-mTOR/mTOR (Figure 5D).

## 4. Discussion

This study characterized the neurochemical effects induced by repeated ECS in adolescent as compared to adult rats while including sex as a biological variable. Our main results demonstrated that ECS improved the survival rate of cells, born in the dentate gryus before treatment (age range of BrdU +cells:12-43 days), although these effects were different for each sex and varied with the age of the animal (adolescent vs. adulthood). Moreover, ECS increased the rate of cell proliferation and neurogenic differentiation at different time points after treatment in adult females, as previously also described for adult males and adolescent rats of both sexes. These time-based regulations of neurogenic markers were accompanied by significant increases in mBDNF protein content, which persisted long after treatment ended. In general, these beneficial effects were more prominent when ECS was administered during adulthood, as compared to adolescence, they normalized over time, and showed similar regulations for both sexes (see combined analysis for both sexes at Supplementary Figures S1, S2). Overall, these data demonstrated the induction of common molecular mechanisms and with similar timelines after treatment taking place in hippocampus after ECS exposure in male and female rats during adolescence and adulthood.

In line with prior results demonstrating that ECS increased hippocampal cell genesis in adult male rats (reviewed by An and Wang, 2022), and together with our prior data that proved increases in cell proliferation and neuronal progenitors in adolescent rats of both sexes and adult male rats (García-Cabrerizo et al., 2020; Ledesma-Corvi and García-Fuster, 2023), the present results provide new data on the long-term survival of cells of different ages at the time of analysis (age range of BrdU +cells:12-43 days) and labeled by BrdU prior to treatment. Note that the absolute number of +cells cannot be directly compared between both sexes, ages and/or days post-treatment, since brains were collected at different experimental waves with particular environmental conditions (e.g., García-Cabrerizo and García-Fuster, 2016). In any case, some age differences should be expected in relation to and/or diluted by the higher basal number of proliferation cells (and therefore labeled) present in adolescence, and as expected given the stage during brain development, in comparison to adult rats. Moreover, long-term survival in adult male rats persisted over time as +cells were aging (1 d: 12-14 days old; 8 d; 19-21 days old; 15 d; 26-28 days old; and 30 d: 41-43 days old), in line with prior results indicating that newly born cells can survive for at least 3 weeks following ECS treatment in male adult rodents (reviewed by An and Wang, 2022). For female adult rats, and adolescent rats, increased BrdU survival was only detected 1-day post-treatment. However, if male and female rats were to be analyzed together and independently of sex, data showed general increases in the rate of long-term survival up to 15-days post-treatment in adulthood, and 1-day post-treatment in adolescence, reinforcing the lower response observed for this age group (see Supplementary Figure S1) in parallel to a shorter antidepressant-like effect (e.g., García-Cabrerizo et al., 2020). As for the possible phenotype of this new surviving cells being incorporated into hippocampus following ECS treatment, one could speculate that most of BrdU +cells would become neurons, since prior studies have proven that this is the case for over 90% of the cells undergoing this process (i.e., García-Fuster et al., 2010), therefore these new cells will likely integrate in the region, become functional and improve plasticity (i.e., synaptogenesis).

Moreover, to better understand how ECS regulated the early stages of hippocampal neurogenesis in relation to biological sex and/or age of treatment, we evaluated cell proliferation (Ki-67 labeling) and neurogenic differentiation (NeuroD labeling) in adult female rats, which together with prior published results in adolescent rats of both sexes, as well as in adult male rats (García-Cabrerizo et al., 2020; Ledesma-Corvi and García-Fuster, 2023) showed sex- and age-similarities in their temporal regulation. In particular, ECS induced an increase in the number of cells undergoing proliferation (i.e., cells born during the last day of treatment) and as measured 1-day post-treatment, for both sexes and age periods of study; this rapid increase was followed by a significant decrease in proliferation rates observed 8-days post-treatment during adolescence, or 15 days post-treatment in adulthood, and a later normalization (only evaluated in adulthood 30-days post-treatment) (Supplementary Figure S1). This course regulation mimics what is typically observed for a repeated treatment followed by removal, in which compensatory adaptative mechanisms, which can even lead to drops in activity, are taking place during removal to reduce the initial treatment effects and restore homeostasis (see for example a similar course regulation of a given marker, increase–decrease-normalization, following a pharmacological treatment with cocaine: acute, repeated and withdrawal phases; García-Fuster et al., 2009). In the present case, one of the compensatory adaptative mechanisms taking place could for example be mediating the inactivation of neural stem cells in response to particular stimuli (e.g., Matsubara et al., 2021). As for neurogenic differentiation, ECS treatment induced significant increases in the number of NeuroD +cells as detected 1- and 8-days post-treatment during adolescence (Supplementary Figure S1; García-Cabrerizo et al., 2020; Ledesma-Corvi and García-Fuster, 2023); while in adulthood increased NeuroD was found up to 15-days post-treatment, with a peak expression observed 8-days post-treatment, to then normalize at 30 days (Supplementary Figure S1).

All our data aligned with prior studies performed mainly in adult male rodents in which ECS robustly triggered hippocampal neurogenesis (Madsen et al., 2000; Malberg et al., 2000; Scott et al., 2000) and increased neural stem cells recruitment and activation (e.g., Segi-Nishida, 2011; reviewed by An and Wang, 2022). However, not much is known regarding whether neurogenesis is necessary for ECS antidepressant-like effects (see Schloesser et al., 2015; Olesen et al., 2017; for prior positive or negative results on this topic). For example, ECS did induce different antidepressant-like effects by sex at the behavioral level and as measured in the forced-swim test (García-Cabrerizo et al., 2020). However, at the neurochemical level, ECS seemed to be inducing similar responses independently of sex (see also previous data: García-Cabrerizo et al., 2020), and/or independently of the dose intensity used to generate the seizures (Ledesma-Corvi and García-Fuster, 2023). Therefore, the molecular effects observed in hippocampus by ECS seemed to be independent of its antidepressant-like efficacy. This is mainly because no efficacy was observed for adolescent or adult females with this specific parameter (95 mA), yet neuroplasticity responses seemed to be in place. Also, when lowering the dose intensity to 55 or 75 mA to induce seizures, an antidepressant-like response was observed in adult female rats, while the effects induced over Ki-67 and NeuroD were of the same magnitude as an inefficacious dose (95 mA; Ledesma-Corvi and García-Fuster, 2023). Moreover, the pharmacological inhibition of basal cell proliferation with temozolomide prevented the antidepressant-like effect of ECS in adult male rats, while only partially blocking the very robust increase in the initial cell markers of hippocampal neurogenesis (García-Cabrerizo et al., 2020). These results suggested that the significant increase in neurogenesis by ECS, apart from having a role in mediating its antidepressant-like effect, might be mediating other neuroplastic actions (García-Cabrerizo et al., 2020). In this context no one has evaluated the exact role for the vast increase in progenitors and cell survival following ECS, and even though an excitatory pro-neurogenic response is usually considered beneficial, the induction of seizures in animal models of epilepsy generates misplaced neurons with abnormal morphological and electrophysiological properties, a process referred to as aberrant neurogenesis (Pineda and Encinas, 2016; Bielefeld et al., 2019).

In addition to the effects induced by ECS on hippocampal neurogenesis, other related molecular partners such as mBDNF or downstream markers (i.e., p-ERK1/2/ERK1/2 and p-mTOR/mTOR, see Figure 1A) were also evaluated. In line with prior reports suggesting increased levels of mBDNF in hippocampus following ECS in adult male rodents (reviewed by An and Wang, 2022), our data extended that knowledge by replicating the data in adult male rats while also proving persistent increases in this neurotrophic factor both during adolescence (up to 8-days post-treatment) and adulthood (up to 30-days post-treatment) in a similar fashion for both sexes (Supplementary Figure S2). These results reinforce the fact that mBDNF is a key transducer common for different antidepressants (e.g., Björkholm and Monteggia, 2016; Casarotto et al., 2021; Madjid et al., 2023) and with a clear contribution to the positive response of ECS (e.g., An and Wang, 2022). Yet, and similarly to what was observed for the neurogenic process, there is a lack of correlation between the course of the behavioral effects induced by ECS (e.g., lack of female efficacy; García-Cabrerizo et al., 2020; Ledesma-Corvi and García-Fuster, 2023) and the neurochemical responses elicited. Finally, for the accompanying partners of mBDNF, the results showed no changes in any of the markers analyzed, except for an unexpected increase in p-ERK1/2/ERK1/2 15 days post-treatment in adult rats, an overall effect for both sexes (Supplementary Figure S2). This could have been speculated to be linked with the down-regulation of cell proliferation observed at this time point in adulthood, however, similar results would have been expected in adolescence at the 8-days’ time point (Supplementary Figures S1, S2).

In terms of the potential limitations of the present study, one could discuss that the effects of ECS in naïve rats will not match those observed in an animal model of psychopathology. However, we proved in a prior study that ECS exerted similar behavioral and neurochemical (i.e., neurogenic markers) responses both in naïve and maternally deprived rats (García-Cabrerizo et al., 2020), a model of early-life stress and initiator of later behavioral alterations (e.g., Marco et al., 2015; Bis-Humbert et al., 2021). Also, ideally, we would have compared both sexes and ages in one given experiment, however the capacity of our breeding colony together with the limited number of animals that could be evaluated at a given time-point, determined the logistics of each experimental wave. Still, when comparing the basal regulation of the molecular markers for SHAM control groups at the different time points of evaluations, similar results were observed within each age and sex, but also generally between sexes of a particular age, suggesting if any, mild variations caused by separate experiments and/or different time points occurred throughout the year (e.g., Supplementary Figure S1).

Overall, repeated ECS increased hippocampal plasticity, by modulating the different stages of neurogenesis in conjunction with mBDNF, at different time points after treatment. Interestingly, most of these effects persisted for a long-time (up to 15- or 30-days post-treatment), longer than the antidepressant-like response characterized at the behavioral level (up to 3-days post-treatment; see García-Cabrerizo et al., 2020), and were parallelly observed for male and female rats. The disparity in the course regulation between the behavioral and neurochemical response could indicate that the initial behavioral effect activated certain plasticity mechanisms that persist past the perceived improvements in affect. Interestingly, in female rats, even when no efficacy was observed following ECS as measured in the forced-swim test (García-Cabrerizo et al., 2020; Ledesma-Corvi and García-Fuster, 2023), still similar molecular events were taking place as the ones observed in male rats in which ECS rendered efficacious. In conclusion, our novel data described similar sex- and age-related changes in neurogenesis and other neuroplasticity markers in hippocampus, as the ones extensively described before for adult male rodents. Therefore, the clinical data regarding ECS safety for adolescence, together with the paralleled molecular events exerted by ECS in adolescence and adulthood (although normally with a shorter effective response in adolescence), suggested comparable treatment outcomes independently of age, in favor of the clinical recommendation of increasing the use of ECT for adolescent treatment-resistant depression. Finally, more preclinical research needs to be performed in female rats to figure out the proper conditions/parameters to elicit efficacy, as well as to better understand the underlying meaning of manifesting similar molecular outcomes following ECT treatment but different behavioral responses as compared to male rats. Another possibility is that maybe ECS does in fact elicit an antidepressant-like response in females, in parallel to the neurochemical responses, but novel scoring methods might be needed to behaviorally quantify it, since the effects were not observed in the forced-swim test. This will require designing new measuring tools to ensure later translation based on biological sex.

## Data availability statement

The raw data supporting the conclusions of this article will be made available by the authors, without undue reservation.

## Ethics statement

The animal study was approved by Local Bioethical Committee (University of the Balearic Islands) and the regional Government (Conselleria Medi Ambient, Agricultura i Pesca, Direcció General Agricultura i Ramaderia, Govern de les Illes Balears). The study was conducted in accordance with the local legislation and institutional requirements.

## Author contributions

SL-C: Conceptualization, Formal analysis, Writing – review & editing, Data curation, Investigation. MG-F: Conceptualization, Formal analysis, Writing – review & editing, Funding acquisition, Supervision, Writing – original draft.
