# Supplementary material for: Electroconvulsive seizures regulate various stages of hippocampal cell genesis and mBDNF at different times after treatment in adolescent and adult rats of both sexes
Source: Front Mol Neurosci. 2023 Oct 30;16:1275783. doi: 10.3389/fnmol.2023.1275783 (PMC10642262; doi:10.3389/fnmol.2023.1275783)
Supplement: Supplementary file 1 [file Data_Sheet_1.PDF]

Supplementary Material

Sex similarities in the time course regulation of hippocampal neurogenesis and neuroplasticity markers by electroconvulsive seizures in adolescent and adult rats

Sandra Ledesma-Corvi S, M. Julia García-Fuster*

*** Correspondence:** M. Julia García-Fuster: j.garcia@uib.es

1 Supplementary Figures

Supplementary Figures include the combined analysis of rats of both sexes for each marker evaluated in hippocampus of adolescent and adult rats by immunohistochemistry (Supplementary Figure 1) or western blot analysis (Supplementary Figure 2). In particular, the present study provided new data on the time course effects of ECS on Ki-67 and NeuroD +cells in adult female rats, which combined with previous published results from our group in adolescent male and female rats and adult male rats (García-Cabrerizo et al., 2020; Ledesma-Corvi and García-Fuster, 2023), suggested similar time course sex-effects induced by ECS (see Supplementary Figure 1). Moreover, Supplementary Figure 2 shows the combined analysis of rats of both sexes for each neurochemical marker evaluated by western blot analysis (mBDNF, p-ERK/ERK and p-mTOR/mTOR).

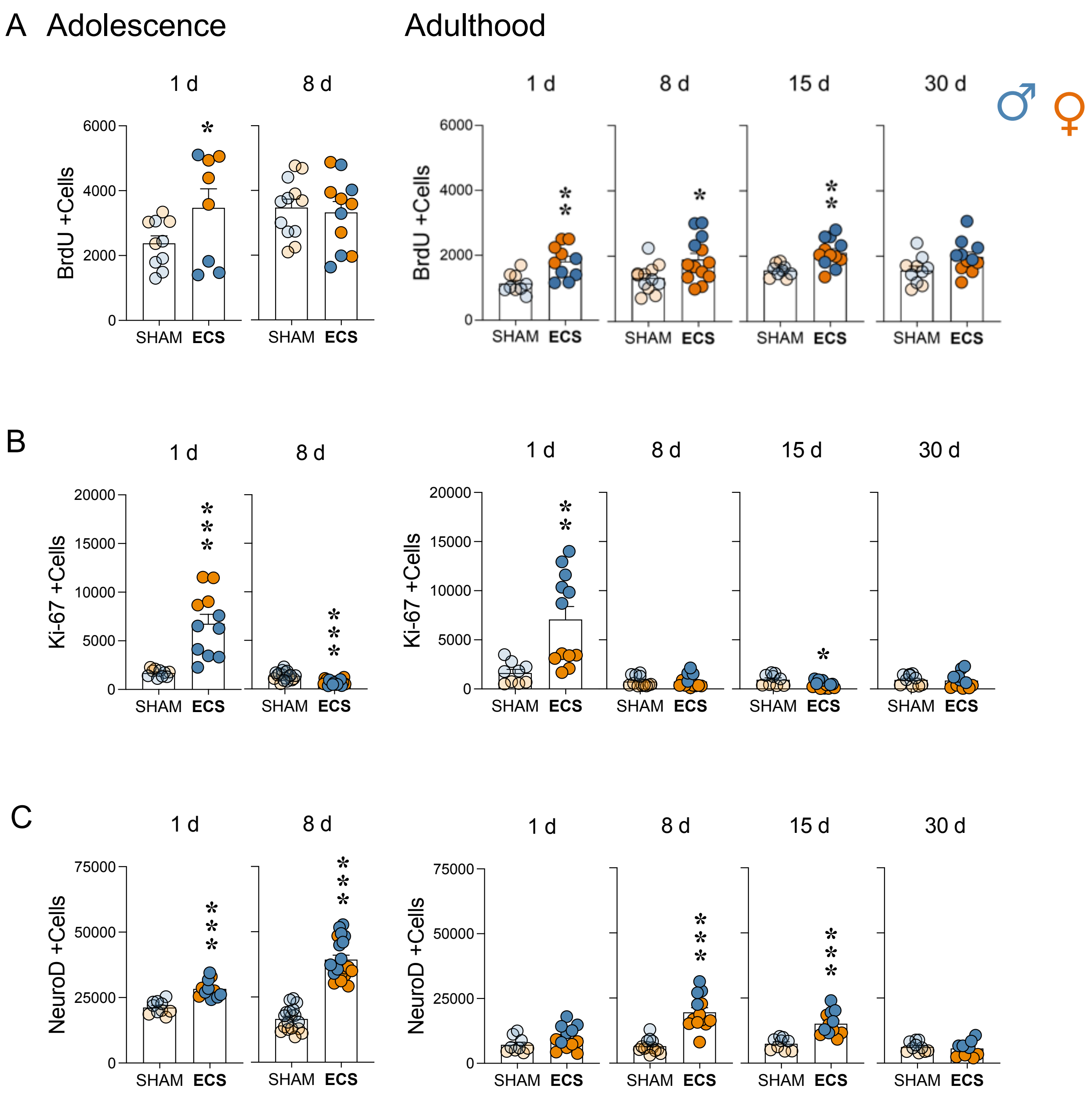

Supplementary Figure 1. Modulation of hippocampal (A) cell survival (BrdU +cells), (B) cell proliferation (Ki-67 +cells) and (C) neural progenitor survival (NeuroD +cells) by repeated ECS treatment in male + female adolescent rats as measured 1-day and 8-days after treatment, or in male + female adult rats as measured 1-, 8-, 15- and up to 30-days post-treatment. Data represent mean ± SEM of the number of total estimated +cells in the dentate gyrus as measured by immunohistochemistry analysis. Individual values are shown for each rat (symbols). Pair comparisons for each sex and age of study were done by Student's *t*-tests: *$p<0.05$, **$p<0.01$ and ***$p<0.001$ vs. SHAM rats. Data for Ki-67 and NeuroD +cells for adolescent male and female rats and for adult male rats were re-analyzed from García-Cabrerizo et al. (2020) and Ledesma-Corvi and García-Fuster (2023), and combined with the data from the current study in adult female rats.

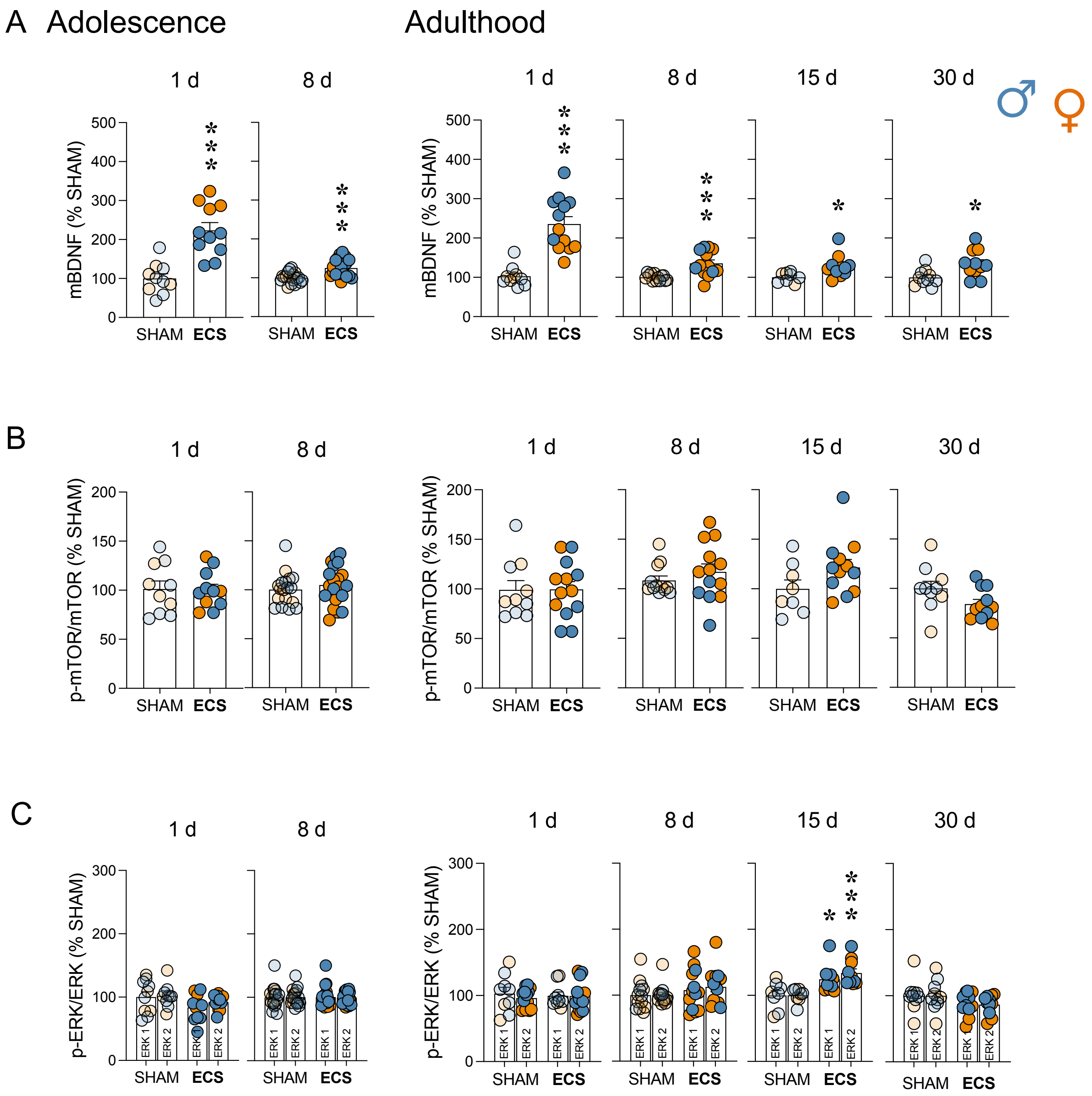

Supplementary Figure 2. Evaluation of the protein content of certain neuroplasticity markers in hippocampus following repeated ECS treatment by western blot analysis. (A) mBDNF (B) p-ERK1/2/ERK1/2 and (C) p-mTOR/mTOR in male + female adolescent rats as measured 1-day and 8-days after treatment, or in male + female adult rats as measured 1-, 8-, 15- and up to 30-days post-treatment. Data represents mean ± SEM of the ratio between the phosphorylated vs. total form of the protein evaluated and expressed as % change vs. SHAM-treated control rats at each particular time. Individual rates are shown for each rat (symbols). Pair comparisons for each sex and age of study were done by Student's *t*-tests: **p*<0.05 and ****p*<0.001 vs. SHAM rats.
